# Internet-Based Cognitive Behavioral Therapy for Psychological Distress Associated With the COVID-19 Pandemic: A Pilot Randomized Controlled Trial

**DOI:** 10.3389/fpsyg.2021.684540

**Published:** 2021-06-14

**Authors:** Victoria Aminoff, Malin Sellén, Elise Sörliden, Mikael Ludvigsson, Matilda Berg, Gerhard Andersson

**Affiliations:** ^1^Department of Behavioural Sciences and Learning, Linköping University, Linköping, Sweden; ^2^Department of Biomedical and Clinical Sciences, Linköping University, Linköping, Sweden; ^3^Department of Psychiatry in Linköping, Linköping University, Linköping, Sweden; ^4^Department of Acute Internal Medicine and Geriatrics in Linköping, Linköping University, Linköping, Sweden; ^5^Department of Health, Medicine and Caring Sciences, Linköping University, Linköping, Sweden; ^6^Department of Clinical Neuroscience, Karolinska Institute, Stockholm, Sweden

**Keywords:** COVID-19, depression, anxiety, internet-based cognitive behavior therapy, controlled trial

## Abstract

**Background**: The COVID-19 pandemic has been associated with various negative psychological consequences. This is a challenge for the society as regular psychological services cannot be offered to the same extent as before the pandemic. In addition to the requirement of social distancing, there is a need to adjust psychological treatment components like exposure to avoid increasing the spread of the infection. Internet-delivered cognitive behavior therapy (ICBT) has an established evidence base for a range of psychiatric problems and has been suggested as one possible approach to deal with the situation. This study aimed to conduct a randomized controlled pilot trial during the summer of 2020 with a broad focus on psychological distress and a treatment approach that tailors the intervention based on symptom profile and preferences.

**Methods**: Following the advertisement and interview, we included 52 participants with elevated levels of psychological distress. They were randomly allocated to either a 7-week-long individually tailored ICBT (*n* = 26) or a wait-list control condition (*n* = 26). Measures of depression and quality of life were used as primary outcomes. We also included secondary outcome measures of anxiety, insomnia, trauma, stress, anger, and alcohol use. For screening, we used the CoRonavIruS Health Impact Survey (CRISIS).

**Results**: Overall moderate to large between-group effects were found at post-treatment in favor of the treatment on measures of both depression [Beck Depression Inventory (BDI); Cohens *d* = 0.63; Patient Health Questionnaire (PHQ-9): *d* = 0.62] and anxiety [Generalized Anxiety Disorder-7-item scale (GAD-7); *d* = 0.82]. This was also observed for stress symptoms [Perceived Stress Scale (PSS-14); *d* = 1.04]. No effects were seen on measures of quality of life, insomnia, symptoms of post-traumatic stress, and anger. There was an effect on alcohol use [Alcohol Use Disorder Identification Test (AUDIT); *d* = 0.54], which was not of clinical relevance.

**Conclusion**: Individually tailored ICBT shows initial promise as a way to reduce psychological problems in association with the COVID-19 pandemic. A possible limitation was that the trial was conducted when the effects of the pandemic were decreasing and when fewer people were affected by the restrictions (e.g., the summer of 2020).

## Introduction

In March 2020, the WHO declared the spread of SARS-CoV-2 as a pandemic (Arden and Chicot, 2020). The related disease, COVID-19, had by then infected more than 1,18,000 people in over 110 countries (Shah et al., 2020). The virus causes respiratory diseases, from a mild to severe degree, and is spread between people (Onyeaka et al., 2020).

A pandemic of an infectious disease affects not only physical but also mental health (Xiao et al., 2020). A global pandemic is likely to lead to fear and worry and influence psychological well-being (Shah et al., 2020). In particular, as a result of being very sudden and highly contagious, the virus causes anxiety, depression, and stress (Horesh and Brown, 2020; Wang et al., 2021). Most of the research on the pandemic has focused on understanding the virus, infection patterns, and physiological symptoms, and in particular to rapidly develop a vaccine. The psychological aspects and suffering have also been focused on, but to a lesser extent (Shah et al., 2020). Given the large costs for society, it has been argued that the indirect negative effects of the pandemic should also be focused on (Wang et al., 2021).

Fear, anxiety, anger, and post-traumatic stress disorder are symptoms that can occur when there is a direct threat of being infected or an indirect threat in response to restrictions in life (Onyeaka et al., 2020). The psychological impact can remain for a long time after the infection, and the spread of the virus is under control (Onyeaka et al., 2020). During the ongoing pandemic, people are urged to keep their distance from each other, isolate themselves, and stay in quarantine. The most fundamental method of dealing with crises, mutual social support, is thereby in some ways not possible (Horesh and Brown, 2020). Quarantine and isolation have been associated with fear (Rubin and Wessley, 2020), acute stress syndrome, depression, post-traumatic stress syndrome, insomnia, irritability, anger, and emotional exhaustion (Jung and Jun, 2020). In the absence of in-person interaction, depression and anxiety tend to occur to a greater extent and even worsen (Xiao, 2020). In turn, stress and anxiety are also associated with reduced sleep quality (Xiao et al., 2020). Quarantine and/or isolation may also lead to boredom, loneliness (Shah et al., 2020; Xiao et al., 2020), and a feeling of losing control (Kessler et al., 2007). Lack of physical activity, in addition to the lack of social interaction, can also lead to increased stress levels (Xiao et al., 2020). In summary, even if individuals who are in quarantine or have isolated themselves have a lower risk for infection and largely maintain their physical well-being, negative psychological effects can emerge (Brooks et al., 2020; Xiao et al., 2020). Moreover, psychological effects seem to be milder when people voluntarily follow the restrictions in comparison with when the restrictions are imposed by authorities (Brooks et al., 2020).

The pandemic may worsen mental health and lead to stress-related problems (Horesh and Brown, 2020). In a study by Wang et al. (2020), half of the participants rated the psychological impact of the virus as moderate to severe, and a third reported moderate to severe anxiety. In a review by Brooks et al. (2020), the authors noted that symptoms of post-traumatic stress disorder, confusion, and anger were the most frequently reported negative effects of quarantine. The impact of quarantine and isolation on a psychological level appears to be extensive and long-lasting, even extending after the quarantine period (Brooks et al., 2020). An associated expected economic downturn has also increased fear and stress (Onyeaka et al., 2020). Many have already faced, or will face, unemployment and economic stress when industries and community services close, which contributes to the negative emotions (Azim et al., 2020). The physical symptoms of the virus, such as cough and fever, can aggravate cognitive suffering and anxiety because of fear of the virus (Shah et al., 2020), and even though a majority of the population may not be affected, media reporting and the experienced risk of becoming infected in the future cause stress and anxiety. Consequently, it is unclear whether the psychological effects are due mostly to medical, social, or economic factors (Horesh and Brown, 2020).

While the WHO and other authorities have focused on the biological and physical aspects (Azim et al., 2020), interventions for mental health in relation to the current situation have been less focused (Shah et al., 2020; Wang et al., 2020; Xiang et al., 2020), even if there have been calls for action (Wind et al., 2020). For both the ongoing work with handling the pandemic and for the parallel work of restoring society following the pandemic, stable mental health is a key factor (Azim et al., 2020; Holmes et al., 2020). Requirements for alternative ways to adapt and work around the crisis come as a result of the need for social distancing (Horesh and Brown, 2020). Internet-delivered cognitive behavior therapy (ICBT) has been investigated and has been found to be effective for a range of psychiatric problems and somatic health problems (Andersson, 2016). ICBT guided by a therapist is as effective as face-to-face treatment (Carlbring et al., 2018) and tends to have larger effects than unguided ICBT-programs (Baumeister et al., 2014). There are several advantages with ICBT in comparison to face-to-face treatment, such as increased availability and cost-effectiveness (Andersson and Titov, 2014).

Individually tailored ICBT has been developed as a way to handle the comorbidity and preferences of the client (Carlbring et al., 2010). Tailoring means that the individual receives different treatment modules depending on symptom presentation, current situation, and preferences (Carlbring et al., 2010; Păsărelu et al., 2017). This means that different clients within a treatment protocol can receive and work with different modules in their treatment. This is in contrast to standardized treatment programs, in which all clients receive the same modules with the same content. Individually tailored ICBT seems to be as effective as standardized internet treatments for depression (Johansson et al., 2012; Kraepelien et al., 2018) and anxiety (Carlbring et al., 2010; Berger et al., 2014; Bergman Nordgren et al., 2014). Choice of treatment modules can be by the therapist in collaboration with the client but can also work when the client makes the selection based on descriptions of the modules (Andersson et al., 2011). It is found that comorbidity between depression and anxiety symptoms is very common (Kessler et al., 2007) and also seems to be the case during the corona pandemic (Wang et al., 2021), and therefore, tailored ICBT treatment could be a way to address this. Psychological interventions, such as CBT, have to be modified to suit the temporary needs of the population during the pandemic. For example, to reduce the risk of infection spreading, CBT should preferably be provided through the internet or telephone. ICBT does not require the presence of a therapist (Wang et al., 2020) and has been proposed as a treatment option during the pandemic (Onyeaka et al., 2020; Wang et al., 2020; Xiang et al., 2020).

The aim of this pilot phase I randomized controlled trial (RCT) was to investigate the effects of individually tailored ICBT with support from a therapist with a focus on psychological symptoms that had occurred or worsened because of the pandemic. Phase I trials are aiming to establish the safety of trials, adverse effects, and information on outcomes by involving small numbers of participants (Umscheid et al., 2011). We addressed symptoms that had developed, or worsened, because of the corona pandemic itself and its consequences, such as social distancing. Except for a trial that found promising effects of a brief ICBT treatment for corona-related worry (Wahlund et al., 2021), this is one of the first trials regarding the effects of ICBT on psychological symptoms linked to the corona pandemic and its consequences.

## Materials and Methods

### Study Design

The study was an RCT in which participants were randomly allocated either a 7-week-long ICBT or a wait-list control condition. Primary and secondary measures (described more in detail below) were administered in conjunction with the recruitment (pre-treatment) and post-treatment. Participants in the control group received the same treatment a few weeks after post-treatment measurement for the treatment group. The Swedish National Ethics Committee approved the study protocol (Dnr 2020-02313).

### Participants and Recruitment

The recruitment began in June 2020. We aimed to recruit at least 60 participants for this pilot phase I investigation to inform a subsequent larger trial, which was planned and registered in clinicaltrials.org. Given the relative ease of conducting online studies and generating a sufficiently powered sample size, we tested the effects of the intervention, which is usually not possible or recommended in smaller pilot studies (Leon et al., 2011). The study was advertised on social media platforms and was also announced in a national newspaper. Interested individuals were directed to the study web page, www.coronacope.se, where information about the study and treatment principles was provided. The registered individuals signed an informed consent sheet online. They were then instructed to complete the screening measures, including sociodemographic questions and the following questionnaires: Beck Depression Inventory (BDI-II), Brunnsviken Brief Quality of Life Scale (BBQ), Patient Health Questionnaire (PHQ-9), Generalized Anxiety Disorder-7-item scale (GAD-7), Alcohol Use Disorder Identification Test (AUDIT), Insomnia Severity Index (ISI), Impact of Events Scale-Revised (IES-R), Perceived Stress Scale (PSS), Dimensions of Anger Reactions (DAR-5), and The CoRonavIruS Health Impact Survey (CRISIS; as shown below for details). By the time the screening was completed, individuals who met the inclusion criteria (as shown below) were called for a clinical telephone interview. The purpose of the interview was to find areas of relevance for each individual to facilitate tailoring of the treatment, as well as to detect potential obstacles for participation (e.g., ongoing substance abuse or being actively suicidal). In case of suicidality or self-harm behavior, the individual was called and advised on how to seek suitable alternative care (in Sweden, health care is tax-funded and does not require special insurance). Other excluded participants were provided with an individual email with reasons for exclusion (mainly that the treatment offered was not suitable for their problems).

**Figure 1:**
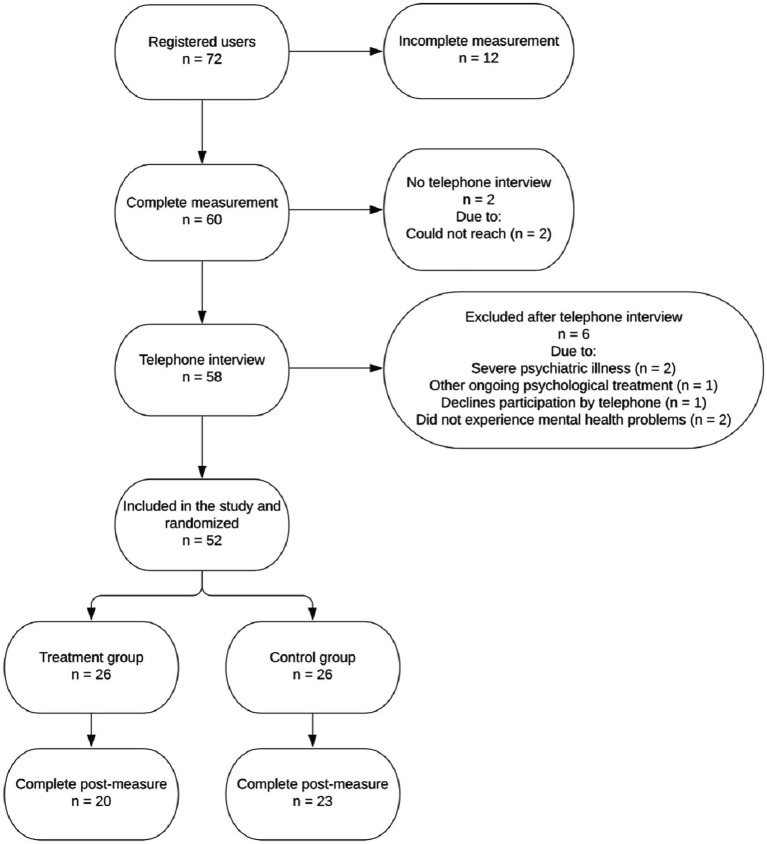
Flowchart of the progress from registration to post-measurement.

In total, 60 individuals completed the screening and were called for a clinical interview. All interviewed cases were discussed during case management meetings with the interviewers (clinical psychologists), a psychiatrist, and the principal investigator, who had the main clinical responsibility. Inclusion and exclusion criteria were discussed, and a decision was made to either include or exclude each participant. Following the exclusion of six persons after the case management meeting, we included 52 participants. These were randomized to either ICBT for 7 weeks (*n* = 26) or to a wait-list control condition (*n* = 26). Randomization was administered through an online random number generator and was performed by a person who was not involved in the research project.^1^ A flowchart of participant progression throughout the study is shown in Figure 1. Descriptive statistics of included participants is shown in Table 1.

**Table 1:** Demographic characteristics of the participants at pre-treatment.

	Treatment (*n* = 26) *n* (%)	Control (*n* = 26) *n* (%)	Total (*n* = 62) *n* (%)
**Age**			
Mean (*SD*) years	42.1 (16.8)	43.4 (18.4)	42.7 (17.4)
Min-Max	22–75	21–78	21–78
**Gender**			
Male	7 (26.9)	8 (30.8)	15 (28.8)
Female	19 (73.1)	18 (69.2)	37 (71.2)
**Highest education level**			
Nine year compulsory school	0 (0)	1 (8.8)	1 (1.9)
Secondary school (completed)	1 (3.8)	3 (11.5)	4 (7.7)
Vocational school (completed)	1 (1.38)	0 (0)	1 (1.9)
College/university (not completed)	7 (26.9)	7 (26.9)	14 (26.9)
College/university (completed)	16 (61.5)	14 (3.8)	30 (57.7)
Other	1 (3.8)	1 (3.8)	2 (3.8)
**Employment status**			
Student	5 (19.2)	5 (19.2)	10 (19.2)
Employed	11 (42.3)	12 (46.2)	23 (44.2)
Unemployed	3 (11.5)	1 (3.8)	4 (7.7)
Retired	2 (7.7)	5 (19.2)	7 (13.5)
Parental leave	0 (0)	2 (7.7)	2 (3.8)
Registered sick	1 (3.8)	0 (0)	1 (1.9)
Other	4 (15.4)	1 (3.8)	5 (9.6)
**Psychological treatment**			
None	5 (19.2)	7 (26.9)	12 (23.1)
Earlier	19 (73.1)	19 (73.1)	38 (73.1)
Ongoing	2 (7.7)	0 (0)	2 (3.8)
**Psychopharmacological medication**			
None	21 (80.8)	23 (88.5)	44 (84.6)
Earlier	3 (11.5)	1 (3.8)	4 (7.7)
Present	2 (7.7)	2 (7.7)	4 (7.7)

### Inclusion Criteria

To be included in the study, participants had to: (1) experience mental health problems and issues which occurred or worsened in conjunction with the pandemic of the coronavirus and/or its consequences, (2) speak, read and write Swedish fluently, (3) have access to a computer, tablet, or a telephone with the internet, and (4) be 18 years or older.

### Exclusion Criteria

If any of the following exclusion criteria were met, the individual was excluded: (1) having a severe mental or somatic illness, which would complicate participation or make participation impossible (including ongoing COVID-19 infection), (2) ongoing alcohol or substance abuse, (3) acute suicidality, (4) ongoing psychological treatment, and (5) having drug treatment with an unstable dosage or a dose, which was planned to change within the time of treatment. The dosage was regarded as stable if it had been the same for the last 3 months. An overall assessment was made on whether acute suicidality was present, including the pre-treatment measures on self-report measures and the information obtained during the clinical telephone interview. Thoughts about death and suicide were, in most cases, not regarded as acute suicidality, but if any plans or more concrete thoughts about course of action were formulated, the individual was encouraged and helped to seek help elsewhere and was excluded from the study.

### Treatment and Therapist

The treatment consisted of seven selected modules out of 16 possible. The participants gained access to one module every week, and the modules were specifically selected based on the answers of the participants on the screening and clinical interview. They also had the opportunity to wish which modules they wanted by the end of the first module. All participants were assigned the first module, Introduction, and the last module, Conclusion, and the maintenance plan. The other five modules were given individually, which dealt with behavioral activation, cognitive techniques, acceptance, emotion regulation, anxiety and exposure, anxiety and worry, anxiety and panic, social anxiety, sleep strategies, perfectionism, stress management, relaxation, problem-solving, and imaginary exposure regarding difficult memories. The modules were derived from a selection of earlier studies (e.g., Carlbring et al., 2010; Johansson et al., 2012) on major depression and anxiety disorder. Modules were adjusted to suit the target group including the mental health problems that may have been influenced by the pandemic and recommendations and restrictions by the Swedish Public Health Agency regarding the spread of the coronavirus. Each module consisted of texts for the participants to read and exercises to work with and write about in the modules. Each participant had a therapist who gave online support and feedback on the work with the modules and exercises and motivated the participant to continue to work with the treatment (Andersson, 2015). Participants were asked to complete the PHQ-9 every week with the purpose to monitor possibly worsening during the treatment. The control group had the opportunity to contact the principal investigator, if needed, and participants were asked to fill in the PHQ-9 every week during the waiting period. The control group was offered an equal treatment of the same procedures as soon as the treatment group had finished (including the post-waiting period for the control group).

The ICBT included support from a therapist, and all communication between the therapists and the participants, except for the clinical interview for inclusion, was conducted through a secure platform. Through this secure platform, self-report measures and the distribution of modules were administered and conducted (Vlaescu et al., 2016). Once a week, the therapists gave feedback on the work of the participants with the modules through text-message option on the platform. At the same time, the therapists gave access to the next coming module. The participants did also have the opportunity to contact their therapist with messages through the treatment platform. If contact was initiated this way, the participant could expect the therapist to answer *via* message within 24 h. The clinicians working with the treatment were one licensed clinical psychologist and three recently graduated clinical psychologists who were experienced in both planning and providing ICBT. The recently graduated psychologists were supervised by the licensed clinical psychologist. The principal investigator was also available for supervision and advice. In addition, a psychiatrist was available during the entire research project and contributed with medical competence and advice.

### Measures

All measures were administered at pre-treatment and post-treatment, except for the CRISIS. The CRISIS is a self-report measure and was developed for covering key domains relevant to mental distress and resilience during the COVID-19 pandemic (Nikolaidis et al., 2020). CRISIS has demonstrated good psychometric attributes, including good construct validity and excellent test–retest reliability, with intra-class correlations between 0.79 and 0.89 in American and British samples (Nikolaidis et al., 2020). The CRISIS was exclusively included in the pre-treatment measure and was the only form of the questionnaires described above that the participants did not receive in the post-treatment measure. The applicants were also asked if they had tested positive for the coronavirus.

#### Primary Outcome Measures

The primary outcome measures were the severity of depressive symptoms, as measured with the BDI-II (Beck et al., 2005), and quality of life, which was measured with the BBQ (Lindner et al., 2016). The BDI-II is one of the most frequently used self-report measures to investigate depressive symptoms (Beck et al., 2005). It consists of 21 depression symptom-related statements, and the individuals estimate how well the statements match their experience. Higher values reflect more severe symptoms. Depressive symptoms are interpreted as minimal (0–13), mild (14–19), moderate (20–28), or severe (29–63; Beck et al., 2005). The BDI-II has good test–retest reliability (*r* = 0.93) and excellent internal consistency (*α* = 0.92) in a clinical population (Beck et al., 2005). The psychometric properties are maintained even when administered on the internet (Holländare et al., 2010).

To examine the quality of life, the BBQ was used, which has 12 statements and the total score ranged from 0 to 96 (Lindner et al., 2016). A higher score indicates a higher experienced quality of life. An optimal cutoff between a clinical and non-clinical group is 52.5, with a sensitivity of 0.79 and a specificity of 0.71 (Frykheden, 2014). The internal and test–retest reliability, as well as the concurrent and convergent validity, are high (Lindner et al., 2016). Lindner et al. (2016) concluded that the BBQ is sensitive to changes and can differentiate between clinical and healthy groups.

#### Secondary Outcome Measures

The PHQ-9, as the BDI-II, intends to measure the degree of depression (Kroenke et al., 2001), and higher values indicate symptoms in the higher extent. It is scored on a range from 0 to 36 based on the answers of the individual on nine items. In addition, the questionnaire includes one question regarding to which extend the depressive symptoms make it harder to handle work, the home, or getting along with other people. Values are assessed as no or minimal depression (0–4), mild depression (5–9), moderate depression (10–14), moderate to severe (15–19), or severe depression (20–27; Kroenke and Spitzer, 2002). Except for having good validity and compliance with clinical assessments (Spitzer et al., 1999), the PHQ-9, by Löwe et al. (2004), has been shown to have excellent internal consistency (*α* = 0.89) and, by Kroenke et al. (2001), also has been shown to have excellent test–retest reliability.

For measuring symptoms of anxiety and worry, the questionnaire GAD-7 was used. It is scored from 0 to 28 and has demonstrated high validity in comparison with clinical assessments and other measures, both in research (Spitzer et al., 2006) and primary care samples (Löwe et al., 2008). Higher scores reflect higher anxiety and worry symptoms. The estimate of an individual on this questionnaire is interpreted as mild (5–10), moderate (11–15), or severe (15 and above). A recommended cutoff when screening for anxiety disorders is 10 (Spitzer et al., 2006). The questionnaire has shown excellent intern consistency (*α* = 0.92) and good test–retest reliability (Spitzer et al., 2006).

The AUDIT consists of 10 items regarding alcohol use, its harmful effects, and dependency symptoms (Berman et al., 2012). When investigated, the AUDIT has consistently shown good validity and reliability. The AUDIT was included as a measure for detecting the level of possible alcohol use and was not primarily analyzed as a separate outcome measure. Higher scores represent a more frequent use of alcohol. Cutoff values for adventurous or harmful use of alcohol are eight for men and six for women (Berman et al., 2012).

The ISI is a questionnaire designed for measuring and investigating symptoms of insomnia, consisting of seven questions and results in a total score ranging between 0 and 28 (Bastien et al., 2001). Higher scores indicate more experienced problems with sleep. These scores are assessed as non-significant insomnia symptoms (0–7), some insomnia symptoms (8–14), moderate insomnia and clinically significant (15–21), or severe and clinically significant insomnia (22–28; Bastien et al., 2001). The instrument has been shown to be a valid and reliable instrument for quantifying perceived insomnia, with a moderate internal consistency (*α* = 0.74; Bastien et al., 2001).

The IES-R is a self-report measure developed to explore symptoms of post-traumatic stress disorder (Arnberg et al., 2014). The questionnaire consists of 22 questions addressing symptoms of re-experiencing, avoidance, and hypervigilance. Weiss (2004) recommends not using cutoff scores for IES-R, but higher scores indicate more severe symptoms of post-traumatic stress disorder. According to Weiss and Marmar (1997), the IES-R has demonstrated high test–retest reliability as well as high internal consistency.

The 14-item self-report measure PSS-14 is designed to detect and measure symptoms of stress and has been shown by Cohen et al. (1983) to have solid psychometric attributes in terms of validity and reliability. The Swedish version of the 14-item version of this questionnaire has not been evaluated in an equally detailed manner, but PSS-10 has shown to have good internal consistency (*α* = 0.84; Nordin and Nordin, 2013). No official cutoff values for clinical significance have been defined.

The brief 5-item instrument DAR-5 is designed to assess the experience of anger (Goulart et al., 2020). Each item is scored on a 5-point Likert scale, which varies from one (none or almost none of the time) to five (all or almost all of the time), with a total score ranging from 5 to 25. Higher values imply the experience of higher anger, and the suggested cutoff value for reflecting psychological distress and functional impairment is 12 (Forbes et al., 2014). According to Goulart et al. (2020), the DAR-5 demonstrates good internal reliability (ranging from *α* = 0.80 to 0.90) and construct validity in general. In their sample, DAR-5 had an acceptable internal consistency (*α* = 0.73).

### Data Analytic Approach

Statistical analyses were performed using IBM Statistics SPSS version 27 (SPSS 27). We used both complete case analysis (CCA) and Intention-to-Treat-analysis (ITT). Multiple regression analysis was used to analyze treatment effects. There are several ways to enter the variables in a regression model. A forced entry was used as recommended by Studenmund and Cassidy (1987). With forced entry, all predictors are forced into the regression model simultaneously. Before multiple regression was performed, assumptions of the linear model were investigated, involving additivity and linearity, independent residuals, homoscedasticity, and normally distributed errors. The assumption of no perfect multicollinearity was added. For all tests of significance, a two-sided approach and the limit, *α* = 0.05, were used.

A broadly recommended method to handle missing data according to Van Ginkel (2020) is multiple imputations (Rubin, 1987). Using this procedure, and by performing 20 imputations, all of the available data were used in the analysis through full information maximum likelihood estimation, and thus, as mentioned above, an ITT approach was applied. This kind of analysis is based on the assumption that data are Missing At Random (MAR). In other words, the probability of missing data is allowed to be dependent on any observed variable but not on the would-be value of the missing data point (Schafer and Graham, 2002).

To calculate effect sizes Cohen’s *d* was used, using adjusted means of post-treatment measures were controlled for pre-treatment measures and observed SDs were controlled for post-treatment measures. Cohen’s *d* is interpreted as 0.20 (small), 0.50 (medium), and >0.80 (large) based on the guidelines by Cohen (1988).

We also calculated reliable change index (RCI), which is a method to compare the values at pre- and post-treatment measures, where the change must achieve a certain level to be considered as reliable when considering the reliability of the measure (Jacobson and Truax, 1991).

## Results

### Sample Characteristics

Descriptive statistics are presented in Table 1. Most of the included participants were female (71.2%), and the mean age was 42.7 years (*SD* = 17.4). Of the 52 participants included in the study, none declared that they had been tested positive for the COVID-19 virus. Using independent *t*-tests, no differences were found between the treatment and control groups regarding any continuous baseline variable (all *p*’s > 0.05) with two exceptions. The treatment group had higher ratings on BDI-II [*t*(50) = −2.06, *p* = 0.044] and PHQ-9 [*t*(50) = 2.12, *p* = 0.039]. Because the pre-treatment measures were used as predictors to explain the variance of the post-treatment measures, this was not considered as a major issue but should be and is taken into account when interpreting the results. The two groups did neither differ regarding age [*t* (50) = 0.24, *p* = 0.81] nor any other demographic characteristics investigated with *χ*^2^-tests.

### Treatment Dropout and Missing Data

Those who did not complete the post-treatment measures were defined as dropouts. As the flowchart in Figure 1 illustrates, data loss at the post-treatment was nine (17%) in total, with six (23%) from the treatment group and three (11.5%) from the control group. Accordingly, 43 (83%) individuals completed the post-treatment measures. Using independent samples *t*-tests, there were no differences on the pre-treatment measures (all *p*’s > 0.05), with the exceptions of GAD [*t*(50) = 2.86, *p* = 0.006] and PSS [*t*(50) = 2.26, *p* = 0.028], with higher scores in the non-completers group. No differences between completers and non-completers were observed regarding demographic characteristics (*χ*^2^-tests). Participants who did not complete the post-treatment and thus were defined as dropouts completed 1.67 (*SD* = 1.37) modules on average. Little’s Missing Completely at Random (MCAR) test was not statistically significant, *χ*^2^(9) = 10.412, *p* = 0.318, which indicates there was no apparent pattern explaining missing data.

### Treatment Adherence and Therapist Time

Treatment adherence was defined when the participant had responded in the treatment platform regarding exercises in the modules or sent a message to the therapist, indicating that the participant had understood the main purpose of the module. On average, participants completed 4.31 (61.6%) out of the seven modules (*SD* = 2.57). Of all 26 participants who were randomized to the treatment group, 10 (38.5%) completed all assigned modules. The module Introduction, which was included in the treatment of all participants, was the most completed module among all other modules with 25 participants out of 26 completing it. After Introduction, the module focusing on cognitive techniques was the second commonly completed module, which was completed 13 times, and the module addressing behavioral activation was the third commonly completed module with 12 participants completing that. The module least completed by the participants was the one about social anxiety, which was completed by zero participants. Then, the three modules about anxiety and exposure, anxiety and panic, and problem-solving were completed by one participant each. In parenthesis, the number of participants who completed each module are as follows: acceptance (9), anxiety and worry (8), stress management (8), emotion regulation (8), sleep strategies (6), relaxation (4), perfectionism (4), and imaginary exposure regarding difficult memories (2). Except for the change in scores of IES-R, pre-treatment and post-treatment scores, and the number of modules completed (*r* = −0.49, *p* = 0.026), treatment adherence was not correlated with change scores of any of the other outcome measures (all *p*’s > 0.05).

The therapists spent on average 111.3 min (*SD* = 67.8) on each participant in total, and the average time spent per week was 15.90 min (*SD* = 9.89). The minimum time spent on a participant was 0 min and the highest was 358 min.

### Analysis of Treatment Effects

#### Main Outcome Measures

Table 2 shows descriptive statistics for each condition at each assessment point with the imputed data. B- and beta-values from the regression analysis are presented, with group condition as a predictor controlling for pre-treatment scores. Standardized regression coefficients and corresponding effect size for each outcome measure at post-treatment measure are reported in Table 3.

**Table 2:** Imputed means, SDs, and the number of participants for each measure divided by condition and assessment point.

Variable	Assessment point	Treatment			Control		
		*mean*	*SD*	*n*	*mean*	*SD*	*n*
BDI-II	Pretreatment	24.62	8.43	26	20.31	6.17	26
	Posttreatment	13.62	11.30	26	16.41	7.19	26
BBQ	Pretreatment	52.54	20.48	26	47.96	20.23	26
	Posttreatment	56.76	22.51	26	50.39	19.16	26
PHQ-9	Pretreatment	11.23	5.19	26	8.65	3.19	26
	Posttreatment	5.83	5.42	26	7.44	4.12	26
GAD-7	Pretreatment	11.46	5.56	26	9.54	3.32	26
	Posttreatment	5.04	4.61	26	7.67	4.53	26
AUDIT	Pretreatment	2.96	3.37	26	4.00	3.64	26
	Posttreatment	2.09	1.92	26	3.76	2.90	26
ISI	Pretreatment	12.42	5.80	26	9.65	5.60	26
	Posttreatment	9.67	5.98	26	8.13	4.46	26
IES-R	Pretreatment	27.73	19.70	26	21.58	15.91	26
	Posttreatment	12.28	13.56	26	17.43	16.18	26
PSS	Pretreatment	35.08	6.90	26	33.35	5.99	26
	Posttreatment	24.86	8.99	26	30.20	6.51	26
DAR-5	Pretreatment	9.50	3.75	26	9.69	2.91	26
	Posttreatment	7.61	2.92	26	8.56	2.96	26

BDI-II, Beck Depression Inventory II; BBQ, Brunnsviken Brief Quality of Life Scale; PHQ-9, Patient Health Questionnaire; GAD-7, Generalized Anxiety Disorder-7-item scale; AUDIT, Alcohol Use Disorder Identification Test; ISI, Insomnia Severity Index; IES-R, Impact of Events Scale – Revised; PSS, Perceived Stress Scale; DAR-5, Dimensions of Anger Reactions.

**Table 3:** Regression model of the impact of group condition on primary and secondary outcome measures with variance explained by pre-treatment measure included.

Variable	Unstandardized coefficients [95% CI]		Standardized coefficients			Between-group effect size [95% CI]
	B	SE B	*β*	*t*	*p*	
BDI-II	−5.48 [−10.53, −0.43]	2.57	−0.29	−2.13	0.03	0.63 [1.18, 0.07]
BBQ	2.86 [−5.29, 10.94]	4.13	0.07	0.68	0.49	0.15 [0.39, −0.70]
PHQ-9	−2.63 [−5.53, 0.28]	1.48	−0.27	−1.78	0.08	0.62 [1.16, 0.05]
GAD-7	−3.55 [−6.11, −1.00]	1.30	−0.38	−2.73	0.01	0.82 [1.37, 0.24]
AUDIT	−1.29 [−2.53, −0.04]	0.63	−0.25	−2.03	0.04	0.54 [−1.09, 0.02]
ISI	0.13 [−2.92, 3.17]	1.54	0.01	0.08	0.93	0.07 [−0.48, 0.61]
IES-R	−6.29 [−14.89, 2.31]	4.38	−0.21	−1.44	0.15	0.45 [1.00, −0.10]
PSS-14	−6.36 [−10.26, −2.45]	1.99	−0.39	−3.20	0.001	1.04 [1.61, 0.45]
DAR-5	−0.72 [−2.02, 0.58]	0.66	−0.12	−1.09	0.27	0.27 [0.81, −0.28]

Between-group effect sizes presented as Cohen’s *d* [95% CI] at post-treatment measure for primary and secondary outcomes, using adjusted means for post-treatment measures with controlling for associated pre-treatment measure and observed SDs.

When controlling for pre-treatment scores, the results from the regression model revealed a significant unstandardized regression coefficient for group condition on BDI-II with *b* = −5.48 95% CI [−0.43, −10.53], *t* = −2.130, *p* = 0.034. This shows that the treatment group, on average, had increased or decreased their estimates on BDI-II at post-treatment measurement, with 5.48 points more than the control group. The between-group effect size was Cohen’s *d* = 0.63 [95% CI: 0.07, 1.18], which is a large effect size. For the BBQ, no significant unstandardized regression coefficient for group condition was found, *b* = 2.86, 95% CI [−5.29, 10.94], *t* = 0.68, *p* = 0.49. The between-group effect size was Cohen’s *d* = 0.15 [95% CI: 0.39, −0.70].

#### Secondary Outcome Measures

No significant effect was found for the group condition as a predictor of the post-treatment outcome on the PHQ-9 when controlling for pre-treatment scores, *b* = −2.63, 95% CI [−5.53, 0.28], *t* = −1.78, *p* = 0.076. The between-group effect size was Cohen’s *d* = 0.62 [95% CI: 0.05, 1.16]. For GAD-7, a significant effect for the group condition was found, *b* = −3.55, 95% CI [−6.11, −1.00], *t* = −2.73, *p* = 0.006, with a large between-group effect size Cohen’s *d* = 0.82 [95% CI: 0.24, 1.37]. For the AUDIT, a significant group effect was found, *b* = −1.29 95% CI [−2.53, −0.04], *t* = −2.035, *p* = 0.043. The between-group effect size was Cohen’s *d* = 0.54 [95% CI: −1.09, 0.02]. For the ISI, no significant effect was found, *b* = 0.13, 95% CI [−2.92, 3.17], *t* = 0.081, *p* = 0.935. The between-group effect size was Cohen’s *d* = 0.07 [95% CI: −0.48, 0.61]. There was no significant effect on the IES-R, *b* = −6.29, 95% CI [−14.89, 2.31], *t* = −1.44, *p* = 0.15. The between-group effect size was Cohen’s *d* = 0.45 [95% CI: −0.10, 1.00]. For the PSS-14, a significant group effect was found, *b* = −6.36, 95% CI [−10.26, −2.45], *t* = −3.20, *p* = 0.001. The between-group effect size was Cohen’s *d* = 1.04 [95% CI: 0.45, 1.61]. For the DAR-5, no significant effect was found for the group condition, *b* = −0.72 95% CI [−2.02, 0.58], *t* = −1.09, *p* = 0.275. The between group effect size was Cohen’s *d* = 0.27 [95% CI: −0.28, 0.81].

The same analyses were calculated with CCA, which gave very similar results as the ITT analyses with imputed data. Data and calculations with CCA are available on request.

### Reliable Change and Negative Effects

Reliable change index was calculated for the BDI-II, GAD-7, and PSS-14. BDI-II and BBQ were the main outcome measures in the study, but since BBQ did not show any significant effects, we calculated RCI for the GAD-7 and PSS-14, which showed statistically significant treatment effects. To calculate RCIs, we used the means of pre-treatment and post-treatment measures of BDI-II, GAD-7, and PSS-14, and SDs for the three scales at pre-treatment measures, using the observed data. For Cronbach’s alpha, values from Beck et al. (2005) for BDI-II (*α* = 0.92) and Spitzer et al. (2006) for GAD-7 (*α* = 0.92) were used. Values from Nordin and Nordin (2013) for Cronbach’s alpha for PSS-10 (*α* = 0.84) were used as this was the most adequate value to use for this calculation. Calculated RCI for BDI-II was 6.09, for GAD-7 was 3.70, and for PSS-14 was 7.06. Missing cases were defined as unchanged.

For the BDI-II, 13 (50%) participants in the treatment group had a reliable change. One (4%) participant showed a reliable deterioration when using the RCI criteria in the other direction. Six (23%) participants did not reach RCI either way, and six (23%) did not complete the post-treatment measure, leaving 12 (46%) participant categorized as unchanged. Regarding the control group, eight (30%) participant reached RCI, and one (4%) participant had reliable deterioration. About 14 (54%) participants were unchanged and 3 (12%) participants did not answer the post-treatment measure, with a total of 17 (66%) participant categorized as unchanged.

For the GAD-7, 11 (42%) individuals in the treatment group reached RCI. None of the individuals showed a reliable deterioration, and nine (35%) did not reach RCI. Together with the six (23%) participants who did not answer the post-treatment measure, 15 (58%) were considered unchanged. About eight (30%) participants in the control group reached RCI, while five (19%) participants were shown to have a reliable deterioration. About 10 (38%) participants did not reach either RCI or deterioration and, as with BDI-II and the other measures, three (12%) participants within the control group did not complete the post-treatment measures, leaving 13 (50%) participants categorized as unchanged.

For the PSS-14, 12 (47%) participants in the treatment group reached RCI. Eight (30%) participants did not reach RCI and none of the participants showed reliable deterioration. Together with the six (23%) dropouts, 14 (53%) participants were considered unchanged. Regarding the control group, five (19%) participants reached RCI, while 18 (69%) participants did not reach RCI. Together with the three (12%) participants who did not answer the post-treatment measure, 21 (81%) participants categorized as unchanged. As in the treatment group, none of the participants showed reliable deterioration.

## Discussion

The present study was designed to investigate the effects of a transdiagnostic individually tailored ICBT for different psychological symptoms, such as depressive symptoms and anxiety, which had occurred and/or worsened because of the corona pandemic and/or its consequences. We prepared the study and report it as a phase I pilot investigation with focus on effects. The treatment modules were adapted to suit the current situation, and overall, the approach worked. We found similar results as reported in previous ICBT studies (Andersson et al., 2019). The results were also in line with a previous ICBT study on corona-related worry (Wahlund et al., 2021). Given the need for safe, effective, and cost-effective internet interventions (Wind et al., 2020), the results are promising and should be followed by a larger trial. Based on the primary outcome measure BDI-II, the intervention resulted in a significantly larger reduction of depressive symptoms compared to the control condition (*d* = 0.63). This result was also observed on the secondary depression outcome measure, the PHQ-9 (*d* = 0.62). The treatment did not show a significant effect on the quality of life, as indicated by the second primary outcome measure, the BBQ (*d* = 0.15). Apart from the PHQ-9, two of the secondary outcomes, the GAD-7 (*d* = 0.82) and the PSS-14 (*d* = 1.04), were statistically significant, indicating that the treatment decreased anxiety symptoms and stress symptoms. We do not regard the effect on the AUDIT (*d* = 0.54) as clinically relevant due to floor effects and differences at baseline. The other three secondary outcome measures, ISI (*d* = 0.07), IES-R (*d* = 0.45), and DAR-5 (*d* = 0.27), did not indicate any significant effects on insomnia, symptoms of post-traumatic stress disorder, and anger.

Tailored ICBT, including support by a therapist, was thus shown to be effective for depressive symptoms with moderate effect size, in line with earlier studies (Sztein et al., 2017; Kraepelien et al., 2018). This was observed on both the primary outcome measure, BDI-II, and the secondary outcome measure, PHQ-9. With regard to reliable change (RCI), 50% of the participants in the treatment group showed a reliable change on the BDI-II. The modules which addressed depressive symptoms contained behavior activation and cognitive restructuring, which are commonly used interventions within CBT for depression. The findings suggest that these components were useful in this setting as well, where the focus was on the pandemic and its consequences. The specific changes that were made in the modules to make them suitable for the pandemic situation were mostly implemented in the examples given in this study. One example is the reinforcing activities in behavioral activation that one can engage themselves with when considering the risk of infection.

We found no effects on the second primary outcome, BBQ, which intends to measure the experienced quality of life (Lindner et al., 2016). The effect of ICBT on quality of life is varied across studies, and not only could the lack of change at least partly depend on the outcome measure BBQ but also that the treatment did not directly focus on the areas that are measured in the BBQ (e.g., friendship, spare time). More specifically, the modules included in the treatment did potentially not address the topics that the participants needed to result in a higher experienced quality of life. The therapists and the participants had 16 modules to choose from and to wish for, whereas other topics, such as loneliness, mindfulness, or working with self-esteem, were not included and may have been more important. Further, experienced quality of life can involve many things, including employment and the private economy, which also could have changed because of the pandemic and its consequences. Finally, it is also possible that the quality of life is a construct that shows lower response to ICBT than to face-to-face treatment (Hofmann et al., 2014).

In contrast to the experienced quality of life, anxiety symptoms as measured with the GAD-7 decreased in the treatment group in comparison to the control group, with a large between-group effect. This was in line with results from earlier studies on ICBT for anxiety disorders (Richards et al., 2015; Andersson et al., 2017, n.d.). In terms of reliable change, 42% of the participants reached these criteria according to the RCI. The anxiety modules essentially contained commonly used CBT strategies for anxiety, such as exposure and cognitive techniques. As with the depression modules, we implemented small adjustments of the previous material in relation to the pandemic and the risk of spread of the virus. For example, an individual with social anxiety was given the example to expose himself or herself to the trigger to call someone instead of going to a shopping mall, which before the pandemic would have been encouraged. In retrospect, anxiety could have been a primary outcome, but this was not what we planned and expected. However, the modules seem to have worked.

The effect on the secondary outcome measures, AUDIT and PSS-14, was also found, both with moderate-to-large effect sizes. The finding that perceived stress, as measured with PSS-14, was reduced mirrors the effects reported in other ICBT trials for stress (Haber et al., 2017). Unlike symptoms of stress, which could be seen as being targeted by the modules in the treatment (e.g., stress management and relaxation), a module specifically dealing with alcohol use was not included. Treatment of anxiety, stress, and depression may have an indirect effect on alcohol use (Riper et al., 2014), but we need to consider floor effects that may not be of clinical relevance.

We did not find significant treatment effects on the ISI, the IES-R, or the DAR-5. The treatment involved modules specifically dealing with insomnia, difficult memories, and emotion regulation, but only a proportion of participants worked with these modules. As the treatment was individually tailored, all participants had their treatment plan with individually combined modules. This explained why insomnia, difficult memories, and feelings of anger did not decrease more. It may also be a question of power and sample size. Earlier studies have reported several negative effects of the pandemic and its consequences on psychological well-being, such as fear (Rubin and Wessley, 2020; Shah et al., 2020), anger (Jung and Jun, 2020), anxiety, depression, and other stress responses (Horesh and Brown, 2020; Wang et al., 2021). These symptoms are not isolated, and comorbidity between depression and anxiety symptoms for example is high (Wang et al., 2021). The choice of an individually tailored ICBT-treatment was therefore motivated but has consequences for the secondary outcome measures and for problems that were not common across participants.

Two main strengths of this study were the use of a controlled design and the adaption of treatment material for the current pandemic. There are also limitations. One limitation is the use of a waiting list as the control condition, which does not control for non-specific factors and result in higher between-group effect sizes in comparison to active controls (Gold et al., 2017). A second limitation is a fairly poor adherence, as the participants in the treatment group completed only 4.31 (61.6%) out of 7 modules. This is somewhat lower than that found in other ICBT studies (Van Ballegooijen et al., 2014). One explanation could be that only a limited number of the modules felt relevant for the participants. The ICBT modules were based on an earlier study and were modified and adapted to the situation with the pandemic, but as discussed in relation to the BBQ, it is possible that other topics and modules would have been needed to address experienced psychological problems in regard to the pandemic. Even though 14 different modules were available, we did not include modules on problems, such as loneliness, that could have been more relevant and important to address. However, our impression was that we could give relevant modules to each individual and that it was an advantage to have different modules to select. Another approach would have been to have a transdiagnostic treatment with the same modules given to all participants (Titov et al., 2011). A third possible limitation was that we, unlike many other studies, conducted the trial during the summer of 2020. During the summer, people are usually less troubled by mild to moderate psychological problems compared to other periods of the year (Ayers et al., 2013). During the summer of 2020, infection rates also went down, and many people in Sweden hoped that the pandemic and its consequences would disappear in the autumn of 2020. Now in 2021, we know that this did not happen and thus we are in the process to conduct the larger trial with adjustments of the modules to make them even more adapted to the pandemic. A fourth possible limitation is that we recruited from the general public *via* advertisements. The participants are likely to differ from patients seen in clinics. On the other hand, a clinic-based study would have been difficult to conduct because of restrictions, and there are indications that patients who receive internet treatment may be representative of the general public rather than regular clinic patients (Titov et al., 2010). The treatment was also developed to be beneficial for individuals from the general public, irrespective of psychiatric status.

A final limitation was that we only included self-report measures. With the form CRISIS, typical physiological symptoms of the COVID-19 virus were investigated, and together with demographic questions, individuals were asked if they had been tested positive for the virus, but no health economic measures were included, such as sick leave, which would have been interesting to investigate in relation to both psychological symptoms and effects of the treatment. None of the participants had, at the time of the pre-treatment, tested positive for COVID-19. Nevertheless, we cannot be certain that none of these participants had had the virus since some infected people do not get any symptoms. Moreover, we cannot be certain that no one was infected during the study. No adverse events and no particular statements about getting sick with symptoms, indicating infection of the coronavirus, was reported.

## Conclusion

The current pilot RCT study provides an initial indication of the possibilities of using an ICBT approach for dealing with the various psychological symptoms associated with the corona pandemic (Holmes et al., 2020). The treatment resulted in moderate to large decrease in measures of depression, anxiety, and stress symptoms. Future studies are needed to investigate the long-term effects and to further adjust treatments for the pandemic situation. The study was implemented in Sweden, where there is high access and use of the internet. Sweden has had one approach regarding restrictions and quarantine, while other countries have had other approaches. The pandemic, by definition, impacts the population all over the world and the effects of tailored ICBT would, therefore, be of relevance to investigate in other countries and contexts, with different levels of both internet access and restrictions given by authorities.

## Data Availability Statement

The raw data supporting the conclusions of this article will be made available by the authors on request, without undue reservation.

## Ethics Statement

The studies involving human participants were reviewed and approved by Etikprövningsmyndigheten Sverige. The patients/participants provided their written informed consent to participate in this study.

## Author Contributions

GA initiated the study and planned it together with MB. VA, MS, ES, and MB conducted the interviews, adapted the treatment for COVID-19, and served as therapists in the trial. ML served as a medical consultant and also participated in intake interview meetings and supervision. VA wrote the manuscript and conducted the analyses together with GA. All authors contributed to the article and approved the submitted version.

### Conflict of Interest

The authors declare that the research was conducted in the absence of any commercial or financial relationships that could be construed as a potential conflict of interest.

## Footnotes

^1^www.random.org
